# The SONIA trial: redefining “better” in the sequencing of CDK4/6 inhibitors for advanced breast cancer

**DOI:** 10.3389/fonc.2026.1830471

**Published:** 2026-05-04

**Authors:** Qinghua Ke, Xiaohong Xie, Shiqiong Zhou

**Affiliations:** Department of Chemoradiotherapy, The First People’s Hospital of Jingzhou, Jingzhou, Hubei, China

**Keywords:** advanced breast cancer, CDK4/6 inhibitors, ctDNA, endocrine therapy, overall survival, treatment sequencing

## Introduction

The management of hormone receptor−positive, ERBB2−negative advanced breast cancer has been fundamentally reshaped by cyclin−dependent kinase 4 and 6 inhibitors (CDK4/6i). However, the optimal timing of their introduction remains a critical clinical question. The SONIA randomized clinical trial, with its updated overall survival (OS) results published in JAMA Oncology (1), directly challenges the prevailing oncologic principle that earlier use of effective therapies invariably yields superior outcomes. This investigator−initiated phase 3 study (2) provides the first randomized comparison of first−line versus second−line CDK4/6i sequencing, offering insights that extend far beyond the specific drug class.

## Key findings and methodological nuance

The trial’s core finding—no OS difference between first−line and second−line CDK4/6i strategies (median OS, 47.9 vs 48.1 months; HR, 0.91; 95% CI, 0.77−1.07) [1]—demands a fundamental reconsideration of how we define “better” in treatment sequencing. While a statistically significant progression−free survival after 2 lines of therapy (PFS2) advantage emerged with longer follow−up (31.9 vs 26.7 months; HR, 0.82; 95% CI, 0.71−0.94), this 5−month PFS2 improvement did not translate into an OS benefit. This dissociation between PFS2 and OS is the trial’s most provocative contribution.

Critical statistical caveat: The SONIA trial was not designed as a non−inferiority study. It was powered for its primary endpoint of PFS2, not for OS non−inferiority or equivalence. Consequently, the absence of a statistically significant OS difference does not establish that the two strategies are equivalent. The 95% confidence interval for the OS hazard ratio (0.77−1.07) includes both a potential 23% reduction in mortality with first−line use and a 7% increase, leaving the possibility of clinically meaningful benefit or harm that the study was underpowered to detect. This limitation must be explicitly acknowledged when interpreting the null OS result. Therefore, throughout this commentary we avoid terms such as “survival−equivalent” or “just as good” in a non−inferiority sense. Rather, the data show no statistically significant difference under the trial’s design, with wide uncertainty. The PFS2 finding, while hypothesis−generating, should be interpreted with caution in the context of the null OS result and the lack of a non−inferiority design.

## Biologic insights and hypothesis−generating signals

What explains this divergence? First, the mandatory crossover design—unique among CDK4/6i registration trials—ensured that 84% of patients in the second−line group ultimately received CDK4/6i, mirroring real−world attrition rates and validating that delayed access does not forfeit treatment opportunity. Second, subsequent therapies, including antibody−drug conjugates (ADCs) and PIK3CA inhibitors, were equally accessible to both arms in the Dutch universal healthcare system. This equity in post−progression treatment access may have normalized OS differences—a critical point for healthcare systems with varying access to these novel, often costly, agents.

The *post hoc* finding of differential benefit by menopausal status (interaction P = .01) is intriguing but must be framed as strictly hypothesis−generating. Premenopausal patients appeared to derive substantial OS benefit from first−line CDK4/6i (HR, 0.53; 95% CI, 0.32−0.87) (1), while postmenopausal patients showed none. However, this finding is confounded by a significant imbalance in visceral disease (66% in the premenopausal first−line group vs. 46% in the second−line group), which precludes definitive conclusions. No prospective validation exists, and the subgroup sample size (n=145) is small. Notably, the biomarker substudy (3) published simultaneously in Nature Medicine identified high baseline circulating tumour DNA (ctDNA) as predictive of PFS2 benefit from first−line therapy, independent of menopausal status. Yet an important nuance must be highlighted: high baseline ctDNA levels are strongly correlated with tumour burden and aggressive biology, making it difficult to disentangle a purely predictive role (i.e., identifying which patients benefit more from upfront CDK4/6i relative to deferred use) from a prognostic effect (i.e., identifying patients with inherently worse outcomes regardless of sequencing). The substudy’s analysis attempted to adjust for known prognostic factors, but residual confounding cannot be excluded. Thus, while ctDNA is a promising candidate, its status as a predictive biomarker for therapeutic sequencing remains to be prospectively validated. This suggests that tumour biology and disease burden, rather than menopausal status per se, may be the primary drivers of the observed signal, further emphasizing the need for prospective validation rather than clinical adoption based on these exploratory analyses.

## Patient perspective: incorporating quality of life and preferences

Health−related quality of life (HRQoL) was comparable between the two sequencing strategies, but this group−level finding masks substantial individual variation in treatment preferences. First−line CDK4/6i combination was associated with 86% of patients experiencing any grade ≥3 adverse event (primarily neutropenia, fatigue, and diarrhoea), whereas deferred use allowed a longer period of endocrine monotherapy with its more favourable toxicity profile. For patients who prioritise avoiding early intensive therapy—for example, those with limited social support, demanding occupational roles, or baseline cytopenias—delayed CDK4/6i offers an option with less immediate treatment burden, albeit without proven non-inferiority. Conversely, patients with high−risk features (visceral crisis, rapid symptom progression, or high ctDNA levels) may value the potential PFS2 advantage and the uncertain OS signal in premenopausal subgroups enough to accept upfront toxicity. Shared decision−making must explicitly weigh these trade−offs: a 5−month PFS2 gain (which may or may not be valued equally by all patients) against an 86% risk of grade ≥3 toxicity in the first−line setting. Future studies should incorporate patient−reported preferences for sequencing strategies using discrete choice experiments to quantify how much toxicity patients are willing to accept for a given PFS2 increment.

## Clinical implications and the question of drug selection

For practising oncologists, three practical implications emerge. First, in postmenopausal patients with low disease burden and endocrine−sensitive cancers, initial endocrine monotherapy with planned crossover to CDK4/6i at progression represents a well−tolerated and cost−effective strategy. However, the available data are insufficient to present this as a strong recommendation; it should be considered a reasonable option after thorough discussion of the uncertainty (the confidence interval is consistent with at most a modest OS difference, but non−inferiority has not been demonstrated). Second, the signal in premenopausal patients—particularly those with high−risk features like visceral disease or high ctDNA levels—suggests upfront CDK4/6i combination may be more valuable, but it must be emphasised that this is based on exploratory analyses with a small sample size (n=145) and confounding factors. No prospective validation exists, and thus it cannot be considered a definitive indication. Third, the 91% palbociclib use, reflecting Dutch reimbursement patterns, limits generalisability to ribociclib and abemaciclib.

Generalisability across drug reimbursement patterns: The SONIA trial’s near−exclusive reliance on palbociclib (91% of patients) is a major limitation for regions where other CDK4/6i dominate. In the United States, ribociclib and abemaciclib are frequently used first−line, supported by trials showing OS benefit (MONALEESA−2, MONARCH 3) that palbociclib did not demonstrate in its registration trials (PALOMA−2). While a large Flatiron Health analysis (4) found no significant OS difference among the three agents in real−world practice, other real−world studies have suggested potential differences in efficacy and toxicity profiles (e.g., abemaciclib’s higher diarrhoea risk but possibly greater CNS penetration). The SONIA results should not be automatically extrapolated to ribociclib or abemaciclib without caution. In healthcare systems where palbociclib is the only reimbursed CDK4/6i (e.g., some European countries, parts of Asia), the deferred strategy is directly applicable. However, in regions with access to ribociclib or abemaciclib, the risk–benefit calculus may differ, and dedicated comparative sequencing trials are needed. No statistical interaction by drug type was observed in SONIA, but the trial was not powered for such subgroup analyses. A recent publication (5) further discusses these differences, providing additional context on the generalisability of sequencing data across CDK4/6 inhibitors.

## Limitations and future directions

The trial’s limitations offer opportunities for future investigation. The use of a 10% ER/PR positivity threshold, while aligned with Dutch and many European guidelines, excludes the 1%−10% low−positive population where endocrine sensitivity may be attenuated, highlighting geographic variation in reporting standards. The 48% prior adjuvant endocrine therapy rate, reflecting Dutch guidelines that often omit endocrine therapy for low−risk stage I disease, may overrepresent endocrine−naive patients compared to US practice. Most importantly, the evolving landscape since trial initiation now includes oral SERDs, capivasertib, and increasingly used ADCs. Whether these agents, when deployed after progression, might differentially interact with prior CDK4/6i exposure remains unknown.

Looking forward, three critical questions must be addressed. First, can we prospectively validate a composite biomarker integrating ctDNA dynamics, menopausal status, and genomic alterations to identify patients requiring immediate versus deferred CDK4/6i? Such validation must clearly distinguish predictive from prognostic effects. Second, how do novel agents integrate into sequencing strategies initiated with or without upfront CDK4/6i? Third, what is the patient’s voice in this decision? HRQoL was comparable, but patient preferences for immediate toxicity (86% experiencing any grade ≥3 event with first−line) versus deferred intensive therapy may vary substantially (6).

## Conclusion

The SONIA trial ultimately redefines “better” from maximal early intervention to optimal lifelong sequencing. Its legacy lies not in disproving CDK4/6i value—these agents remain indispensable—but in demonstrating that thoughtfully delayed introduction, guided by biology and clinical context, can preserve outcomes while reducing toxicity and cost. However, this conclusion must be tempered by the trial’s statistical design: the absence of a non−inferiority framework means we cannot claim equivalence, only that no superiority was detected. For postmenopausal patients with low disease burden, deferred CDK4/6i is a reasonable, patient−centred option after thorough discussion of the uncertainty—but not a strong recommendation. As the pharmacopoeia for ER−positive breast cancer expands exponentially, SONIA provides a methodological template: future trials moving drugs from later to earlier lines must mandate crossover, prioritise OS and PFS2 as primary endpoints, rigorously document post−progression care, and be explicitly designed for non−inferiority if equivalence is the intended claim. In an era of multiple effective agents, the question is no longer “now or never?” but “now or later?”—and SONIA reminds us that for many patients, later may be just as good, but we need better trials to prove it.
